# An investigation into the deep learning approach in sentimental analysis using graph-based theories

**DOI:** 10.1371/journal.pone.0260761

**Published:** 2021-12-02

**Authors:** Mohamed Kentour, Joan Lu

**Affiliations:** School of Computing and Engineering, University of Huddersfield, Huddersfield, West- Yorkshire, United Kingdom; Vellore Institute of Technology: VIT University, INDIA

## Abstract

Sentiment analysis is a branch of natural language analytics that aims to correlate what is expressed which comes normally within unstructured format with what is believed and learnt. Several attempts have tried to address this gap (i.e., Naive Bayes, RNN, LSTM, word embedding, etc.), even though the deep learning models achieved high performance, their generative process remains a “black-box” and not fully disclosed due to the high dimensional feature and the non-deterministic weights assignment. Meanwhile, graphs are becoming more popular when modeling complex systems while being traceable and understood. Here, we reveal that a good trade-off transparency and efficiency could be achieved with a Deep Neural Network by exploring the Credit Assignment Paths theory. To this end, we propose a novel algorithm which alleviates the features’ extraction mechanism and attributes an importance level of selected neurons by applying a deterministic edge/node embeddings with attention scores on the input unit and backward path respectively. We experiment on the Twitter Health News dataset were the model has been extended to approach different approximations (tweet/aspect and tweets’ source levels, frequency, polarity/subjectivity), it was also transparent and traceable. Moreover, results of comparing with four recent models on same data corpus for tweets analysis showed a rapid convergence with an overall accuracy of ≈83% and 94% of correctly identified true positive sentiments. Therefore, weights can be ideally assigned to specific active features by following the proposed method. As opposite to other compared works, the inferred features are conditioned through the users’ preferences (i.e., frequency degree) and via the activation’s derivatives (i.e., reject feature if not scored). Future direction will address the inductive aspect of graph embeddings to include dynamic graph structures and expand the model resiliency by considering other datasets like SemEval task7, covid-19 tweets, etc.

## Introduction

Due to the tremendous covering and standardization of social media and Internet of Things on our daily life [1, 2] people feel more confident to consider this digital connected world as a new communication tool. Research in Machine Learning (ML) has widely addressed different ways to assess people’s thoughts and retrieve meaningful correlations to best quantify them, this is known as Sentiment Analysis (SA). The latter has revolutionized several domains by considering users’ understanding and feedback about specific topics to improve their trustworthiness and therefore benefits businesses [3], this includes:

- Business: assessing customers’ voices [4], market research and analytics [5] (e.g., e-business), reputation management [6], etc.
- Technology: Recommendation systems [7], robots’ adaptation [8], assessing astronauts’ mental health [9], etc.
- Social actions: Real world events’ monitoring, smart transport/cities [10], social media monitoring (i.e., racism detection [11, 12]), etc.
- Politic: peaceful government solutions [13], clarifying politicians’ positions, opinions inversion prediction [14], etc.
- Healthcare: approaching people from different background/races by extracting common feedbacks and correlations [15], retrieving insights in order to improve treatments (e.g., breast cancer treatment experience [16], brain data [17] has been extracted to infer correlations among naïve speakers, etc).

Most of these works perceived SA as a classification task (e.g., Support Vector Machine (SVM) [18], Naïve Bayes (NB) [19], bias impact on ML [20], etc.). In this sense, recent works have shown promising outcomes by boosting the performance of these algorithms. In [21], a feature selection mechanism has been proposed and outperforms some classical selection techniques (e.g., Term-frequency, Chi-square, etc.) by providing more context to the feature’s size reduction rather than frequency (i.e., data spread, output correlation, etc.).

Despite some promising classifiers (e.g., NB with 94.02% accuracy [22], SVM and NB with 90% and 95% respectively [23], etc.) in the domains like healthcare for instance, it is known that data (e.g., Functional rehabilitation) are highly correlated [24] and not equally distributed [25]. Those latter exclusions require more better analytic frameworks that merges both computational power and a covering knowledge in order to adjust the SA to the medical field. In this sense, graph generation techniques are known for their expressiveness and deep data processing [26] which gave a way to a recent analysis technology known as graph embedding [27]. The latter technique has been subject to many ML improvements (e.g., reducing input size and feature selection for an accurate text classification [22, 23], etc.).

Latest efforts on Deep Learning (DL) have been showing good function approximations rather than traditional ML ones [28] by using additional components (i.e., thresholds, weights, activation functions, etc.); however, SA for healthcare implies a deep investigation at several levels, that was justified in [29] by using an accompanied text investigation along with the Convolutional Neural Network (CNN) algorithm, which means DL still lacks an extensible feature learning mechanism to best answer the SA process as advocated. In this work, we investigate a new deep neural network method for SA which better approximates the different aspects of SA (i.e., polarity, subjectivity, frequency of terms/tweets within text, etc.), this contribution is two-fold: 1) improving the feedforward path by proposing an embedding strategy for the input unit which reduces the data training complexity within a low-dimensional space. 2) increasing the backward path’s precision by scoring the features following their importance (i.e., high frequency, better activation function approximation, etc.), which guarantees a rapid learning surge with a good performance (i.e., high accuracy, F-score, etc.). Furthermore, the model has been shown to be transparent and efficient.

The remainder of this paper is organized as follows: Section 2 lists the research questions and a set of respective hypotheses which emphasize the developed aspects of this research. Our aims and objectives are detailed in section 3. Section 4 presents the literature review and the theoretical aspect of this research. Whereas, our proposed methods are presented in section 5, this is followed by an experimental study in section 6. We evaluate our model in section 7, and then we critically discussed the whole work in section 8. Section 9 concludes the paper and gives few perspectives.

## Motivation

The mechanism of the actual Deep Neural Network (DNN) has been officially proposed by [30] as a supervised Multi-Layer Perceptron (MLP). To our best knowledge, the same authors were the first introducers of DNNs transparency by training each layer independently and learning their correlated representations. This was a feed-forward model of multiple layers (called connected components) of non-linear activation functions. However, the theory of the input’s influence on the output performance within neural networks was discussed few years before by [31] known as the problem of Credit Assignment Paths (CAPs). The latter consists of deciding which DNN components are influencing the model performance. While this problem could be addressed in a different manner, similar works agreed on the final performance as the main criteria to justify the model’s efficiency. In [32], authors have been investigating the stability of DNN (i.e., multidirectional LSTM) components modelled as a grid as a way to stop DL model vanishing problem. Although authors in [32] have achieved state-of-the-art performance, the complexity of the input space and the state activation layer in [32] remains an issue if deployed with limited resources.

Nowadays, with the emergence of Neuroscience and artificial neural networks [33], CAPs are not only limited to a certain layer. Moreover, back-propagation strategy [34] remains inefficient in certain vanishing or overfitting problems, which are more likely to occur due to the equal consideration of the input samples (see [21]).

As SA became popular for many DL applications, the lack of transparency in decision making within specialized domains like medicine [35] is quite misleading and some practices may oppose to the General Data Protection (GDPR). To our best knowledge, CAPs has not yet been investigated in this research area whereas it was the origin of DL transparency as stated before. Therefore, by this research, we aim to investigate CAPs theory for a transparent DNN structure that best answers the SA. In contrast to the DL models from literature, we want to keep the complexity (i.e., special/temporal, see “Complexity analysis”) as lower as possible, and this will be done by acting on the building cycles of a DNN (i.e., feedforward and backward paths) while restricting the input features in a lower space representation and then scoring the derivative instances with a selection mechanism respectively.

## Research questions and hypotheses

In order to best understand the proposed research investigation as well as the objective method, the following questions listed in Table 1 aim to frame this research into the right context. A set of hypotheses have been proposed followed each research question.

**Table 1:** The proposed research and the following hypotheses.

Index	Research question	Hypothesis
RQ1	How can DL models be transparent?	Applying graph embeddings on the DNN’s input unit may give a better view on the importance degree of the input neurons.
RQ2	Are explainable DL techniques adequate with feed-forward and back-propagation?	Preserving the activation functions and the backpropagation algorithm complies with the feedforward and backward paths respectively.
RQ3	Graphs are meant to be visually easy and understandable, does that apply to Graph Neural Networks (GNNs)?	Extracting a vector features from graph embedding may clarify the way how features are selected in a DNN, which is still very challenging [36].
RQ4	Which and how structural units of DNNs could be optimized with compliance to their working mechanism and theory behind?	Defining the input centrality weights may provide further predictive insights if matched with the embedded vector.
RQ5	What is the state of the art of DL on SA? And how does that fit with the proposed graph based explainable method?	Merging graph embedding with attention learning may increase the model efficiency.

## Aims and objectives

**Fig 1:**
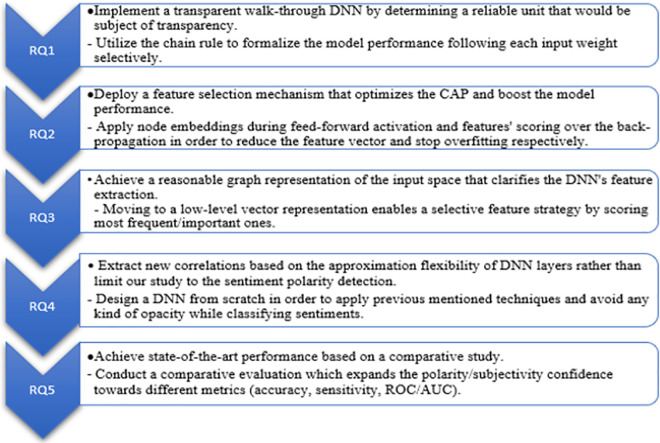
The proposed aims and objectives.

Only few attempts have tried to associate graph technologies to the deep sentiment analysis process [37, 38]. The aim of the proposed method is to study the influence of the input nodes and hidden layers on the final DNNs outputs, in such way, getting the right sample features will help to reduce the features vector space while keeping the model rationality. This was inspired from the attention mechanism [39] along with deploying the deep neural architecture. The study will focus on people’s tweets, the goal is to enrich the DNN structure with graph embedding learning [27], which will be refined through a selective strategy. The following Fig 1 associates each proposed research question with the envisaged aims and objectives respectively.

As shown in Fig 1, we aim for each research question to be answered following the associated objectives, and that for the following purpose:

1. Answering that question will help to emphasize the increasing trend toward explainable DL and the different approaches (see “Transparency in DL”).
2. Expending this question allows to figure out a convenient way to abstract a given DL problem while being rational to the internal structure (see “Abstraction strategy”).
3. By exploring this question, most recent GNNs have been reviewed and the main obstacle for making them understandable was highlighted (see “Graph based neural networks”).
4. This question will help to reveal a partitioning method that permits to identify the DNNs unit concerned by the proposed method (see “Methods”) and that has impact on the whole performance.
5. This question will motivate the most recent attentional mechanism within SA and the way to merge that with graph embeddings methods (see “DL applications on SA”).

## Literature review

In this section, we review most recent applications of DL on SA and their performance. Then, we address explanability within DL by emphasizing recent graph-based learning models.

### Research strategy

The following strategy denotes the main resources and the data extraction scheme which allows a good reflection of the multidimensionality topic of DNNs with respect to the SA field. This is followed by an evolution chronology and a careful combination of the topics’ components (CAPs, graphs, SA, DL) which together motivate the proposed method.

#### Literature resources

**Fig 2:**
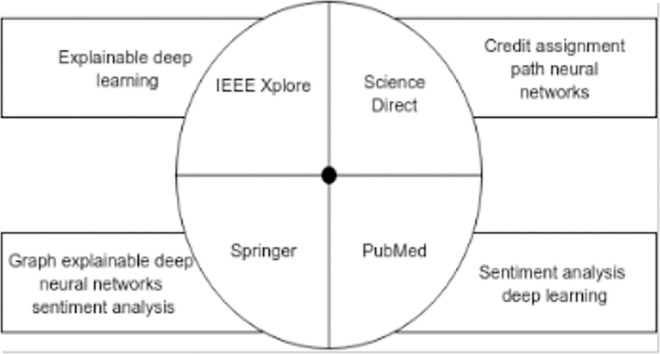
Research databases and keywords.

**Fig 3:**
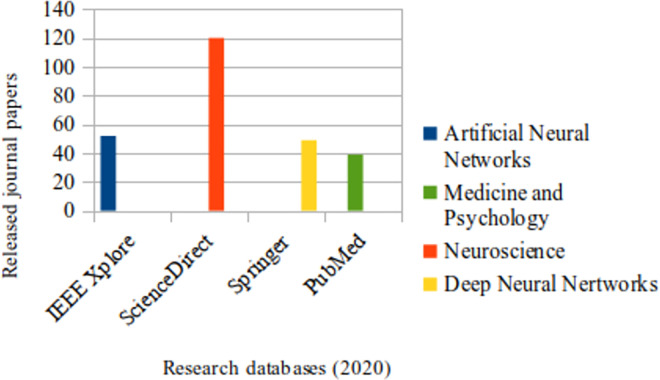
Released papers for each database corresponding to each related subject.

**Fig 4:**
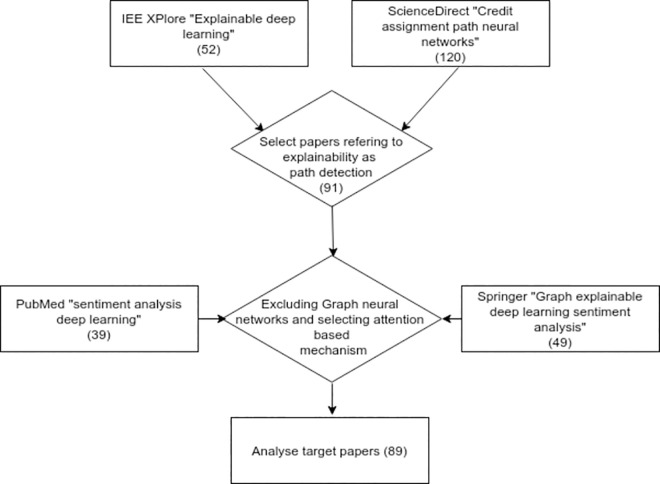
Journal papers selection method.

IEE Xplore, ScienceDirect and Springer research databases were invoked in order to retrieve papers from journals which refer to explainable DL, journal papers referring to SA have been reviewed from PubMed database, this has been refined to include works based on DL in particular. The context and key words related to each database as well as the selection results are illustrated in Figs 2 and 3 respectively, whereas the following diagram summarizes the selection strategy (Fig 4).

#### Subject evolution

**Fig 5:**
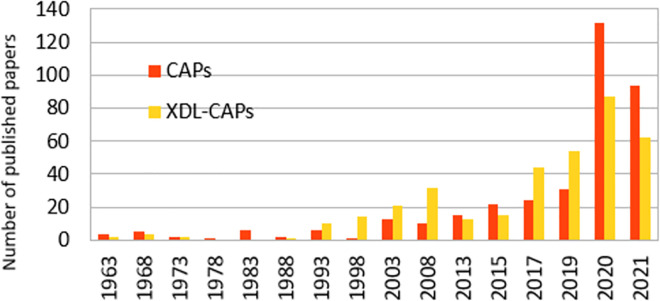
Published papers referring to CAPs and explainable DL with reference to CAPs.

*CAPs and explainable DL*. CAPs is a historical problem [40] which explores causal paths starting from adjusting input’s weights to an optimal output. The majority of works on graph explainable DL have addressed CAPs problem from specific angles, usually referred to as “model specific” [41]; however, only few attempts have tried to position a DNN as a compositional unit [42] and the best way to assign input values which refers to the historic CAPs. As shown in Fig 5, CAPs is gaining more and more attention during last years, as well as published papers with a reference to explainable DL (XDL) and CAPs. Most of them were bio-inspired which treat credits as electric signals coming from external sensors, known as “cause-affect” strategy.

**Fig 6:**
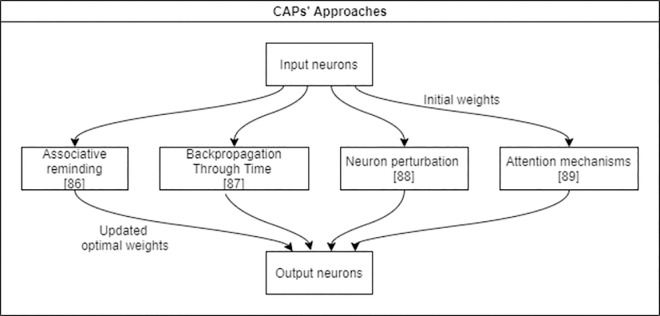
Recent approaches for DNNs credit assignment.

*Graphs and CAPs*. As stated before, research on CAPs has begun as a way to assign credits to better minimize the error function [42]. Fig 6 illustrates new categorization of CAPs’ approaches based on neuron paths’ detection.

The main question which was preventing CAPs from being widely explored as an efficient performance parameter was “whether the brain backpropagates or not”; in this sense, graphs have been subject of research in order to represent the relevance between data patterns [43], RNNs have been firstly proposed to deal with backpropagation, then LSTMs [44, 45] and Sliced RNNs (SRNNs) [46] for a constant vanishing prevention and long term dependencies respectively.

**Fig 7:**
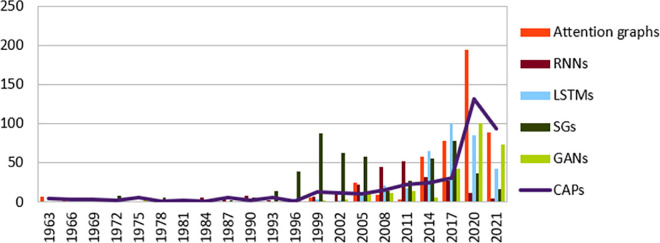
DNNs models distribution over years as a graph based solution to CAPs.

As shown by Fig 7, new models became popular, they’re all characterized by their graphic nature which not only try to solve a learning problem, but to learn how the resolution is inferred [47]. Stochastic learning Graphs (SGs) [48] for instance introduces new gradient setting to best reduce the loss.

Moreover, Generative Adversarial Networks (GANs) have been proving their efficiency in transferable learning by revealing generic analysis patterns [49]. However, large “discrete” graphs (e.g., Multi-hidden DNN) due to discrete independent weights. Furthermore, Attention layers have extended DNN structure [39] (AGs) with an importance degree of nodes or links which alleviate the discrete learning to be inductive with less computation (i.e., without matrix-factorization).

Reinforcement Learning (RL) was the most targeted model while dealing with CAPs, because the way neurons’ weights are updated (by assigning a final weight to a certain neuron) is very similar to the concept of failure/reward within RL followed by seeking an explanation for the result.

### Sentiment analysis

**Fig 8:**
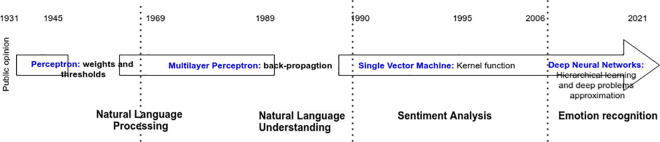
Brief chronology of SA following the development of DL.

SA has becoming a basic-block unit for many modern platforms; its evolution has seen various changes and appellations [50] along with the technology and analytics used for the analysis. Fig 8 represents a progress bar of SA according to neural networks evolution. DL has revolutionized the way SA is conducted, starting from a single perceptron that only supports a limit number of weights and bias, to a relatively better approximation of functions with Multi-Layer Perceptron (MLP) and the introduction of back-propagation algorithm. By mid 90s, SA became very popular by the introduction of kernel functions and Human-interface machines known as “Brain Computer Interface”.

As certain admit that emotion detection is the future trend of SA [51], the latter is still dominating the field of medicine and psychology where DL is playing a key role on transforming people’ sentiments into computational aspects.

#### Sentiment analysis through CAPs

**Fig 9:**
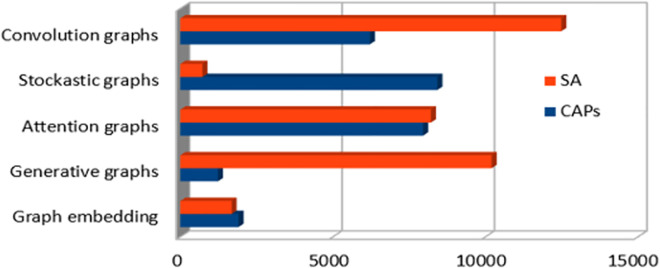
Similar research addressing “SA” and “CAPs” relative to graph technologies between 2000–2021 (based on the previous analysis (Fig 7), graphs have been getting more attention by year 2000).

As modern SA process may imply dealing with long text frames and guarantee inner or outer document dependency, this will initially refer to assigning certain documents to pre-training stage; therefore, it can be subject of CAPs in order to figure out the right parameters. For our knowledge, the latter problem has not been addressed from a CAPs viewpoint yet; However, as shown by Fig 9, it was remarkably shown a similar interest on both graph embedding and attention mechanisms which reflect the effectiveness of graphs in those research areas in terms of selectively highlighting the active set of neurons which can be optimized and the ones which may impact the predicted sentiment in both CAPs and SA respectively.

#### DL applications on SA

SA [52] has proven its ability to retrieve human’s feelings from several confusing texts. However, long term dependency is one of the DNNs’ application limits on SA, which consists of preserving a traceable execution of the model [53]. As a possible answer to the first part of “Research questions” (RQ5), recent models from the literature (Table 2) tried to address that issue by hybridizing some models, like LSTM with GCN [38] for instance; however, a mechanism that detects important patterns is much more needed with source variant datasets, not only for improving accuracy, but for the learning visibility.

**Table 2:** Works on DL for SA.

Level of analysis	Author(s)	DL model	Datasets	Technique	Accuracy
Document based SA	[54]	Bidirectional Long Short-Term Memory Network (LSTM)	TripAdvisor & BeerAdvocate datasets	• Provide an attentional based document hierarchy that preserves both word and sentence semantic as well as the attentional aspect of sentiment.	81%
	[55]	Sentence representation LSTM	Internet Movie DB (IMDB) [56]	• Handle variable sentence lengths and semantic with Glove	44.3%
				• Use LSTM for long term dependencies	
	[54]	Bidirectional LSTM	• TripAdvisor	• Document’s sentences encoding and words’ vector embedding strategy.	70.5%
			• Yelp2014 [57]		
	[56]	Discourse-LSTM	• “Movie reviews from rotten tomatoes”	• Discourse-based tree construction using “rethorical theory”	80% on movie reviews 85.2% on IMDb
			• “IMDb movie reviews”	• Perform “bottom-up” approach to capture the overall semantic of the tree.	77.1% on “Amazon reviews”
			• “Amazon fine food reviews”		
Sentence based SA	[29]	CNN	Health-care reform (HCR), Stanford, Michigan, IMDB and Semantic Evaluation of Systems challenge (SemEval) Datasets	• Employ a graph technique to cover stop- words, position, etc., for sentences meaning.	85.71% on HCR
				• Use of CNN for document selection and perform sentiment classification.	83.71% on Stanford
					98.41% on Michigan
					87.69 on SemEval
					86.07 on IMDB
	[58]	Cloud based neural network	• SemEval2014-Task 9	• Handle data deluge complexity by deploying sentiments via web servers using Stand-ford strategy	86.92%
				• Use SVM or NB to classify the sentiment ranking.	
	[59]	• Bidirectional Gated Recurrent Unit	• Yelp-2013	• Slicing the input data with sliced Recurrent Neural Networks (RNNs).	68.04% with Yelp-2013
		• Sliced Recurrent Network	• Yelp-2015	• Perform bidirectional sentences semantic dependency through a fully connected neural network.	74.12% with Yelp-2015
Aspect based SA	[60]	• Bidirectional LSTM,	• SemEval-2014[Table-fn t002fn001] datasets (restaurant and laptop)	• Embed input words as vectors.	82.95% with restaurant review.
		• Graph convolutional network and Bidirectional attention mechanism		• Use Bi-LSTM to return the context of each word’s vector	75.55% with Laptop reviews.
				• Aggregate each node with their neighbour using GCN.	
	[60]	Attention based LSTM	• Korean news articles	• Sentences decomposition using “Word2Vec”	91.28% with Wikipedia
			• Wikipedia, Korean language	• Apply LSTM on each sentence (word-vector) instead of single aspects, in order to get the right context	92.91% with cosmetic’s feedback
			• Cosmetic’s reviews		92.07% with articles
	[61]	Lexicon enhanced attention network	• SemEval2014-task4[Table-fn t002fn002]	• Getting a deep sentence’ meaning by using Bi-LSTM (parallel content aspect representation with sentence’ embedding)	79.1% with restaurant dataset
					73.7% with laptop-dataset
	[59]	Syntax and knowledge base graph convolutional network	• Restaurant-14 7	• Syntactic split of a sentence to build common-sense relationship between words.	83.48% with Restaurant-14 7
			• Laptop-14 7	• Knowledge-graph convolution for tree aspects classification.	75.19% with Lptp-14
			• Restaurant-15[Table-fn t002fn003]		83.20% with Restaurant-15
			• Restaurant-16[Table-fn t002fn004]		87.19% with Restaurant-16
Cross modal SA	[62]	CNN + Dynamic CNN	• ImageNet for CNN [63]	• AlexNet-based CNN is used to extract features during feed-forward propagation.	69.5%
			• Microblog dataset or the whole model		
				• Couple DCNN with Word2Vec algorithm to perform a textual learning with word embedding efficiency.	
	[64]	CNN + Bidirectional LSTM	• SemEval Task-4	• Extract terms’ categories using Bi-LSTM then classify them as polar sentiments using CNN.	88.91% with Task-4
			• SemEval task5		76.42% with Task-5
			• SemEval task-12		65.97% with Task-12
	[65]	• Bidirectional RNN	CMI MOSI [66]	• Encode different data modalities into vector features.	78.05%
		• Attention-based networks.		• Use Bidirectional RNN to capture both direction word’s dependencies.	
		• RNN (Gated recurrent unit, LSTM, Group LSTM-based RNN)		• Attention networks help achieve sort of input node’s importance.	
				• GRU was used to fix the define a standard input for the last SoftMax activation function.	
	[67]	CNN and Pretrained CNNs for transferable learning.	• Construction of a Basic balanced Music-Emotion video dataset.	• Splitting input data with 1-D CNN for pre-processing	88.56%
				• Merge different dimension CNN to the final Soft-max decision function as predictive task.	
	[68]	DL-Multi Level Parallel _Attention Neural network	IMDB movies dataset [57]	• Embedding strategy applies on different SA level.	96.13%
				• Generate and merge attention-based vectors to get prediction.	

^1^ http://alt.qcri.org/semeval2014/task4.

^2^ http://alt.qcri.org/semeval2015/task12/.

^3^ http://alt.qcri.org/semeval2016/task5/.

^4^ http://pan.baidu.com/s/1i4BfrAd.

#### Transparency in DL

There has been a lot of research about clarifying DNNs and whether understanding the internal connection of neurons could improve the model performance [69]. Imaging is one of the emerging fields in DL, the majority of works tried to explain imaging systems from specific problems [70, 71]. However, language processing accompanied with the availability of large text dataset became centre of interest to many researchers, one remarkable work was done by [72] for huge text corpus explanation; although the imaging system is more clarified and flexible, the way the graph was generated doesn’t benefit from graph-based technologies that optimize the input starting from naive generation.

Overall, explanability in DL can be categorized into:

1. **Example-based approaches**; research in this area is always conducted through a training-example, by specifying some initial observations which will be verified through features’ extraction, this discipline is widely adapted despite the difficulty of verifying the trustworthiness of each example, this covers:
  ✓ Gradient methods (e.g., Guided-back propagation, Layer-wise relevance propagation [72]), which aim to a better gradient optimization.
  ✓ Saliency-feature map [73] for measuring pattern importance within images and videos.
2. **Model-based approaches,** which concentrate on the raw data, they’re usually referred to as input optimizers. Some recent works include the pre-processing stage of DARPA [74] where the explainable interface is built on users’ psychological aspect. [75] have explored the fusional aspect of DNNs which aims to “mimic” a function aggregator using fuzzy network, etc.

#### Graph based neural networks

Graphs are playing a crucial role in processing data and preserving their semantics [76]. The idea of combining graph technologies and DL is not recent [77]. As a proof of that, many graph manipulations have been introduced: graph-pooling [78], graph-attention networks [39], etc.

**Fig 10:**
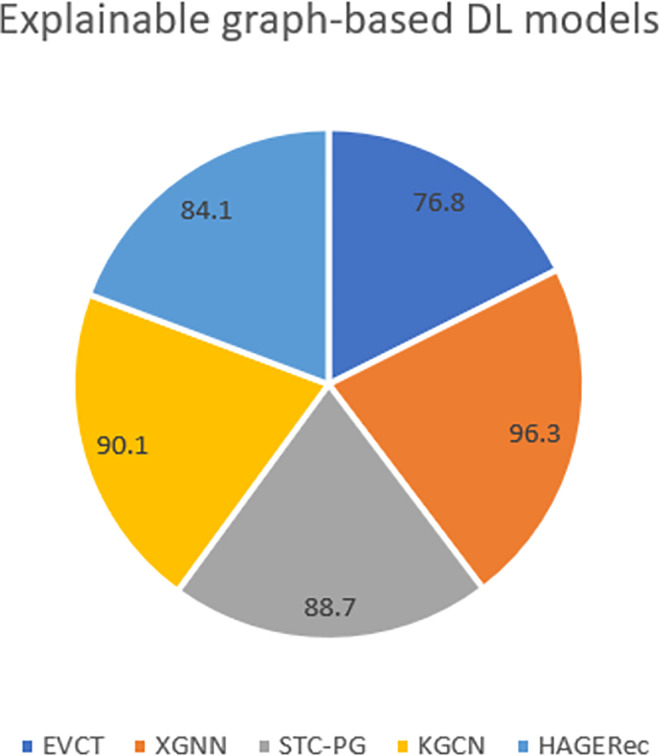
Overall comparison of predictive accuracy. EVCT [72]: Explainable and Visualizing CNN for text information. XGNN [73]: Explainable Graph Neural Network. STC-PG [75]: Spatial Temporal and Causal -Parse Graph. KGCN [76]: Graph-based Convolutional Network for chemical structure. HAGERec [77]: Hierarchical Attention Graph Convolutional Network Incorporating Knowledge Graph for Explainable Recommendation.

However, few attempts have coupled labelled graph generation with a deep learning model apart from the activation function, which makes them extremely hard to explain or to interpret. Fig 10 compares few recent works on graph explainable DL.

The main obstacle of abstracting every single unit of a deep neural network (see “Abstraction strategy”) as a graph structure is the non-compliance with back-propagation process. The work done by [75] is a proof of that where they had to create a function aggregator that simulates the true Choquet-integral mechanism, because graphs could be encoded as adjency-matrix for the best; and that does not fit with the back-propagator as a function optimizer. As an answer to Research questions (RQ3), we investigate recent efforts (Fig 10) and within the below sub-section, in order to retrieve certain limits on GNNs and motivate a model-based approach on the input unit of the DNN.

#### Analysis and discussion on graph-based SA

**Fig 11:**
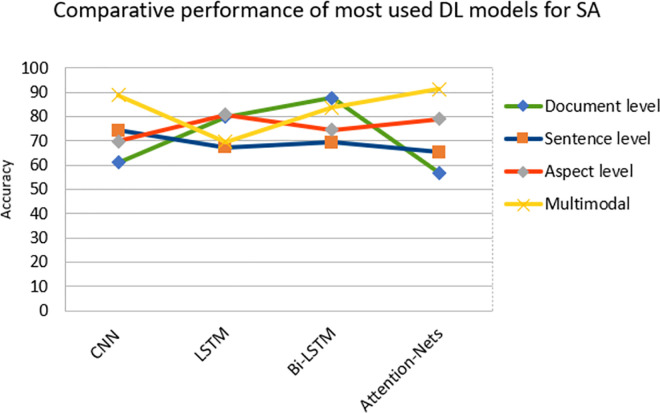
Most used DL models in SA and their accuracy.

The conducted evaluation illustrated by (Fig 11) depicts most DL structures and their variations in terms of accuracy following each analysis level (see 11). When considering documents as a whole, LSTM-based approaches were crucial and showed good performance to capture inter/intra documents’ correlations. However, as long as we move further from sentence-based to a single aspect level, there is much interest on aspects embedding with attention networks, the latter were able to gather neighbourhood context for better sentiment classification. That could be noticed in a recent multi-modal trends’ analysis [67], where RNN and LSTM fail to capture emotions’ boundary for the whole video while Attention-based CNN showed good performance (see Table 2).

The following notes express few limits of recent works on this area:

- GNNs (e.g., Graph attention networks, Attention graphs, Stochastic graphs) (Fig 7) are widely considered in the area of connected data, but large labelled graphs still represent an issue due to their exponential growth, therefore moving from high dimensionality to low space representation is conditioned by being discriminative to the raw data parameters.
- Transferable learning which consists of generalizing the DL model from a specific observation to other domains still an issue to many DL models, because they are built on a specific dataset(s). However, as justified by [79, 80] a further approach could be performed by setting up an input mechanism that map the complexity of raw data to smaller frames while being expressive.
- High dimensional feature analysis remains an issue for most dependency-based models (LSTM [80], GRU [59]); some solutions have been deployed like skip data connections [81] to reduce the input size, they may prevent some vanishing cases, but they add more complexity as additional hidden layers to the gradient. This is why majority of research is now turning to address the agnostic aspect of the explanation, in order to impose a standard limit for the input.

The previous argumentations fall into the example-based approach (see 17), where a model selection starts from an observed fact, like neighbourhood aggregation, short term dependency, etc. However, these methods neglect the impact of DL input units on the performance, thing that justifies the “accuracy” paradoxes (Fig 11) even though a sentence or an aspect may reflect a similar sentiment. Therefore, the challenge will be to provide an explainable solution to the DNN input unit (i.e., model-based approach (see “Transparency in DL”)) as an answer to the “Research questions” (RQ1), which satisfies the CAPs (Fig 9), and this is based on the current research trend (Fig 7).

## Methods

As the healthcare domain is known to be critic and full of complicated scenarios that do not forgive mistakes, one accurate way to perform a deep learning technique is by preserving the model rationality [82]. Although model oriented [83] and example-based approaches [84] have shown an explainable independency level and an input dependent optimization respectively, they both position the problem of clarifying DNNs within a barrier of high interpretability but low accuracy, and vice versa. The proposed approach in this paper consists of designing a novel DNN based on a hybrid graph embeddings/attention scoring.

DNNs are known to provide high accurate outcomes, this is known as the model performance. Formally it is described as:

$$P=\frac{1}{N}\cdot {\left\|d-z\right\|}^{2}\;where:$$

- N is the number of input and hidden layers
- d is the desired output and z is the actual output

**Fig 12:**
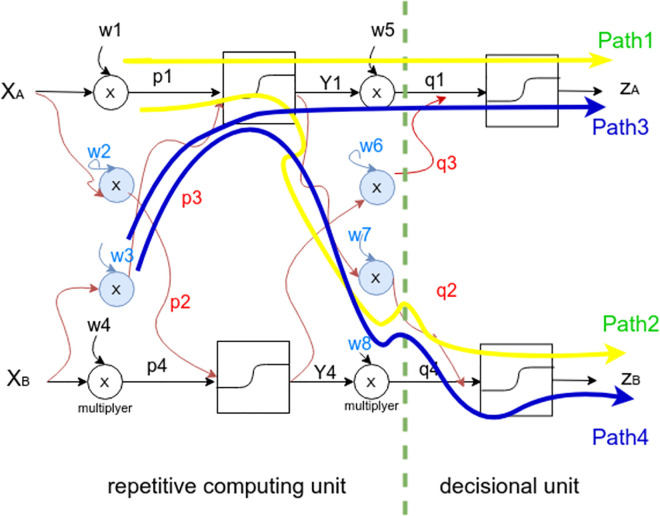
Two hidden layer DNN structure.

Mathematically, the output generation (z) through the feed-forward and back-propagation cycles is expressed as a serie of partial derivatives [33]. For instance, suppose the following in-depth view of a deep neural architecture (Fig 12) which is composed of two hidden layers, two inputs (XA, XB) and two outputs (ZA, ZB).

### Abstraction strategy

In order to answer research question (RQ2) (see “Research questions”) and following the structure depicted by Fig 12, we will explore the impact of the performance “P” on the internal DNN structure. By considering both weights “w1” and “w3”, this could be expressed by the chain rule (1) and (2). The purpose is to justify a structural unit of the DNN model that could be optimized with compliance to the DNN feedforward and backward paths, see (Research questions (RQ4)).


$$\frac{\delta P}{\delta w1}=\frac{\delta P}{\delta Za}\cdot \frac{\delta Za}{\delta q1}\cdot \frac{\delta q1}{\delta Y1}\cdot \frac{\delta Y1}{\delta p1}\cdot \frac{\delta p1}{\delta w1}+\frac{\delta P}{\delta Zb}\cdot \frac{\delta Zb}{\delta q2}\cdot \frac{\delta q2}{\delta Y1}\cdot \frac{\delta Y1}{\delta p1}\cdot \frac{\delta p1}{\delta w1}$$ (1)

$$\frac{\delta P}{\delta w3}=\frac{\delta P}{\delta Za}\cdot \frac{\delta Za}{\delta q1}\cdot \frac{\delta q1}{\delta Y1}\cdot \frac{\delta Y1}{\delta p3}\cdot \frac{\delta p3}{\delta w3}+\frac{\delta P}{\delta Zb}\cdot \frac{\delta Zb}{\delta q2}\cdot \frac{\delta q2}{\delta Y1}\cdot \frac{\delta Y1}{\delta p3}\cdot \frac{\delta p3}{\delta w3}$$ (2)

- It is noticeable that the selected partial derivative units are equal with respect to both “w1” and “w3” and this will be the same for the units with respect to “w2” and “w4”. That refers to the repetitive unit (Fig 12), which means it has no direct impact on the global performance as opposite to the decisional unit, where:
  - the last multiplayer *Y*1⊗*w*5 gives *q*1 as an input toward the activation function and generates *Za* as both Path1 or Path3.
  - However, it is observed that *Y*1 is also implied to generate *Zb* but this time from the multiplayer *Y*1⊗*w*7 and gives *q*2 to the second activation function which forms Path2 or Path4.

So, as much as we move further to the input, there are more computational units which are reused.

**Problem**.

1. Both Inputs “Xa” and “Xb” participate for an intermediate component “Y1” which has an impact on the final model performance.
2. Find a way to establish an importance degree between model inputs (e.g., “Xa” and “Xb”) to figure out the one(s) with higher impact on the final output.

### Input space embedding

Embeddings on graphs are known to be very useful in dealing with huge graph data and random distribution [85]. Suppose G(N, E) a graph of N nodes and E edges, where: E ∈ [1… m] and N ∈ [1… n].

The mapping function is based on a threshold which analyses the neighbourhood connections of each node, suppose (n = 500) is a maximum allowed connection:

In case of node embeddings, for a node n1 with c1 connections:

Map = {N}, f1∈N and c1 < = 500;

or Map = {N—f1} where c1 > 500.

**Fig 13:**
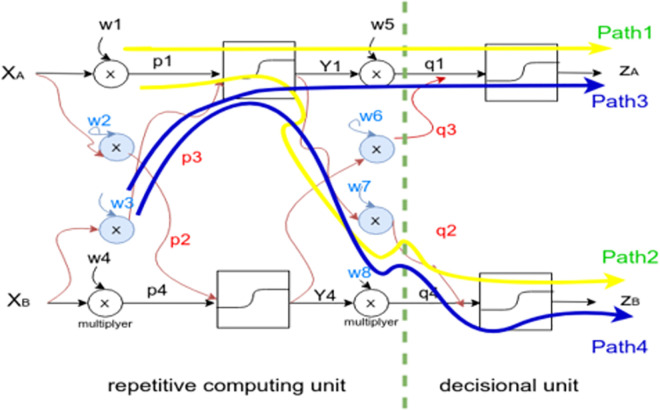
The proposed mode for SA.

The proposed model depicted by Fig 13, consists of a graph-based strategy which aims to reduce the input repetitive unit into a low-level space representation, then into a small vector unit which may alleviate the computation complexity of the whole DNN model.

### Features’ selection via attention scoring

Instead of moving from the embedded vector space (see [23]) through the activation functions, it has been considered to score the embedded features (*v*_1_… *v*_*n*_) following each hidden layer (*L*_1_…*L*_*k*_) with a set of weights *a*_*w*_, w = [1.. n].


$$Scor{\left(i\right)}^{t}=f(L(i-1),v(i),a_{w}^{t-1})$$ (3)

The score vector represents a trace of reaching features, the latter will be mainly envisaged by the back-propagation loss function optimizer (see algorithm below), therefore by considering the activation function ((4) is the” SoftMax” for instance), the attention weight *a*_*w*_(*i*) for a hidden layer (t) will be calculated as following:

$$a_{w}{\left(i\right)}^{t}=\frac{{exp}^{\left(Scor{\left(i\right)}^{t}\right)}}{\sum_{1}^{n}{exp}^{\left(Scor{\left(i\right)}^{t}\right)}}$$ (4)

Starting from the embedded distribution of features, the “Gaussian” distance metric [86] has been considered to score similar (close) features and therefore to generate a “decorated” neural path through the “SoftMax” function for instance and repeatedly to achieve best distribution. A level of genericity is aimed to be reassured in terms of the activation function selection as well as the embedded feature vector. To summarize, the corresponding learning algorithm will be:

**Algorithm:** To implement the proposed DNN mode (Embedding and scoring)

1. **Input:**.txt files //raw dataset

2. **Output:** sentiment-polarity

3. Procedure SA

4. Graph_SA = Networkx_Upload (path to the csv_file)

5. Samples Initializing

6. vect = Embedding (Graph_SA) /*this call may be node/edge embedding*/

7.    FOR each feature within vect do

8.        Input[**x**] = feature

9.        FOR all **x** in DNN do

10.            Output[**x**] = module.forwardPropagation(Input[**x**])

11.            IF Output[**x**] > = threshold /*threshold could be maximum node connectivity(e.g., most frequent aspects*/

12.                Scored[**x**] = Output[**x**] //the selected feature

13.          End

14.         Input[**x+1**] **=** Output[**x**]

15.        End

    /*Activation function condition (e.g., Positive sentiment polarity and attention weights calculation (2) */

16.    Sentiment-polarity = condition(Scored)

17.    IF still training then

18.        FOR each [**k-x**] Scored feature in DNN do //k is the total features’ number

19.            Scored = module.BackwardPropagation /*Backpropagation will stop if feature is not scored*/

20.            Input[**x+1**] = Scored[**x**]

21.        End

22.    End

End

The algorithm above can be explained in three main parts:

- The graph generation and the embedded vector extraction (see “Input Space Embedding”), this covers line 1 to the 10^th^ of the algorithm. The forward activation function is applied for each embedded feature.
- The conditional step which is variant according to a specific domain (e.g., most frequent feature in our case), this corresponds to the line 11.
- The features’ scoring, which a conditional step as well. However, it differs from the previous one as each feature is conditioned with the activation functions’ requirements (i.e., approximation, limit values, polarity, etc.).

### Solution for high dimensional space

Our proposed mode (check the number of models with names of each mode) focuses on the input unit of the DNN, where it has been shown through the chain rule (1) and (2) that any input stream (Fig 12) follows a specific decisional path with respect to the features’ weights. Our case study (see “Experiments”) imposes a 2-d dimensional representation which corresponds to the “station-polarity” prediction. This has been achieved through a graph generation with a neighbourhood embeddings. Therefore, most influential nodes within a given station are the ones having minimal Gaussian distance (i.e., polarity of the most frequent term within the text.).

However, certain DL tasks like time series [87], adversarial examples [88] require an extension of the classical closeness methods (i.e., Gaussian distance), as the data may be distributed within k-dimensional space. Following the graph embeddings strategy denoted previously, a solution to the multidimensional space must satisfy a number of criteria:

- The resulting embedded structure must show a reduced feature sample than the original input one.
- The embedding function must comply with the activation function in order to cope with the path decoration.
- A similar process (i.e., embeddings and scoring) needs to be ensured within the k-dimensional space in order to preserve the output semantic.

The projection of the above criteria results on the mapping probability [89] of a feature’s instance *x*_*i*_ in a *layer*_*i*_ with its respective pattern *x*_*j*_ on a *layer*_*j*_. A higher probability *P*_*i*|*j*_ means a closer instance i from j (i.e., station-polarity in our case):

$$P_{i|j}≝\frac{{exp}^{\left(\frac{{-||x_{i}-x_{j}||}^{2}}{2o^{2}}\right)}}{\sum_{i\neq k}{exp}^{\left(\frac{{-||x_{i}-x_{k}||}^{2}}{2o^{2}}\right)}}$$ (5)

Therefore, by considering all the k-dimensional space, the scoring function (3) as well as the activation function (4), the output attention weight *a*_*w*_(*i*) for a layer (t) will be given by:

$$a_{w}{\left(i\right)}^{t}=\frac{{exp}^{\left(Scor{\left(\frac{{-||x_{i}-x_{j}||}^{2}}{2o^{2}}\right)}^{t}\right)}}{\sum_{i\neq k}\sum_{1}^{n}{exp}^{\left(Scor{\left(\frac{{-||x_{i}-x_{j}||}^{2}}{2o^{2}}\right)}^{t}\right)}}$$ (6)

There is a clear match between the resulting scoring function (6) and the activation function (i.e., SoftMax for instance), and that confirms the second part of “Research questions” (RQ5) on the compliance of the feedforward path with the backward one, which enables an efficient performance (see “Improving DNN performance via a deterministic backward walk”).

## Experiments

In this section, a number of empirical experiments have been applied on tweets HN-datasets (see 27), data has been collected and unified from 16 different health news sources (stations), the proposed SA model goes beyond polarity detection of people’s feedback to the most influential aspects and sentences which contribute to polarity and subjectivity variations.

After data has been cleaned and pre-processed, we aim to build a predictive analysis around most influential tokens among tweets, after that we show the role of edge embedding in terms of transparency and the benefit of visualizing the polarity distribution on a reduced plan.

### Datasets

Health news tweets datasets (HN-datasets) [90] consists of 16 different sources of people’s tweets having experienced or have been exposed to healthcare situation. Data sources are represented through different text files (i.e., goodhealth.txt, foxnewshealth.txt, cnnhealth.txt, etc.), which contain more than 58000 instances and 25000 attributes. The following Table 3 lists some features of “Kaiser Health news”, “Fox news” and “Good Health” stations for instance.

**Table 3:** Characteristics of three health tweets datasets.

Features Station	Number of tweets	Tweets’ encoding	Size (KB)	Overall sentiment
Kaiser health news	3509	Utf-8	3509	positive
Fox news health	2000	Cp1252	2000	positive
Good health	12000	Utf-8	12000	negative

These datasets are used to prove the model working strategy. It has been decided to use these datasets to deal with heterogeneous data (i.e., different encoding, insignificant words, healthcare domain specifications) and perform a global SA of tweets.

### Development environment

This work has been done on a UNIX system (Ubuntu Kylin ver. 20.10, architecture x86_64, processor intel core i5). Python 3.8 was the main programming language adopted for implementing the data procedures and the following data analysis tasks (see next sub-sections in the current section “Experiments”). Jupyter was the main development API with some of the following python libraries for basic functions and visualizations:

- The “glob” module as a Unix pathname style for datasets uploading.
- “nltk” as a natural language toolkit for stop words remover for instance.
- “re” module to deal with the unstructured tweets’ files as regular expressions.
- “math” library to invoke mathematical functions (e.g., “Tanh”, “exp” functions to implement the DNN activations, “log” function for loss simulation, etc.).
- “WorldCloud” library for frequent tokens display.
- “Networkx” for graph generation, etc.

### Data cleaning and pre-processing

The challenging aspect about retrieving tweets from different sources is the heterogeneous nature of data that consists of different encoding styles (utf-8, cp1252, etc., see Table 3), because an overall SA around specific data sources is aimed to be achieved.

#### Text split

As tweets are totally informal, a list of special characters [。?、~@#¥%……&*();:\s+\.\!\/_,$%^*(+\"\’]+|[+―!] has been considered to split lines into raw sequences of tweets containing only natural language terms.

#### Stop word remover

Tweets within the above dataset come with unstructured textual format, therefore a proper tweets analysis consists of splitting sentences/aspects and removing all sort of non-significance in order to retrieve the most meaningful sentiment. NLTK’s stop list English words has been used with more domain specific non-relevant words (i.e., new, may, com, etc.).

### Statistical sentiment analysis

**Fig 14:**
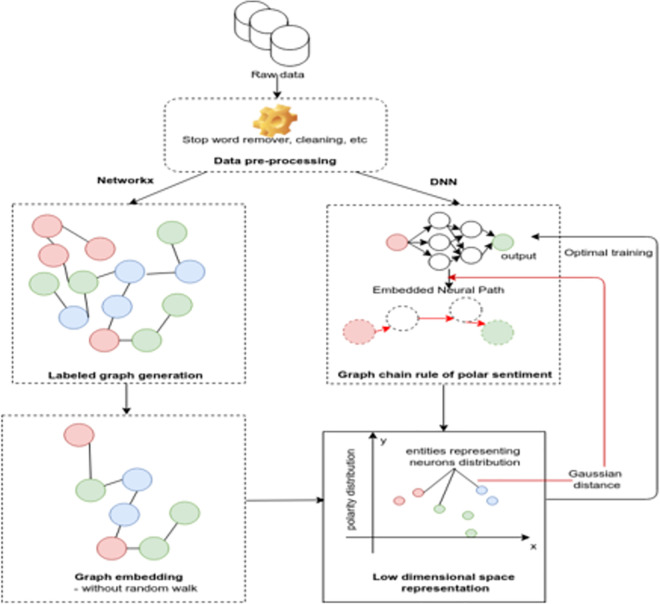
“evcerydayhealth” top-10.

**Fig 15:**
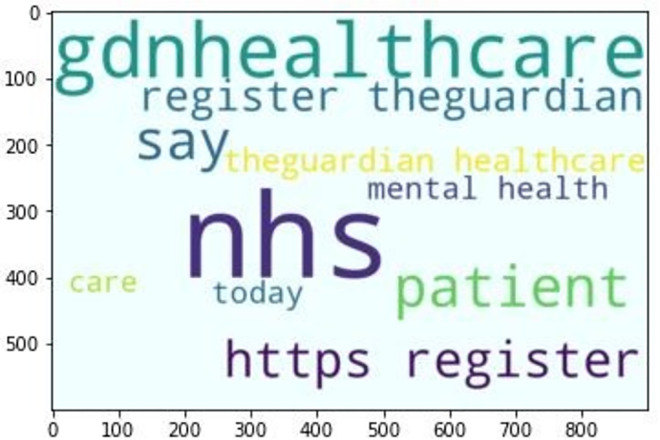
“gdnhealthcare” top-10.

**Fig 16:**
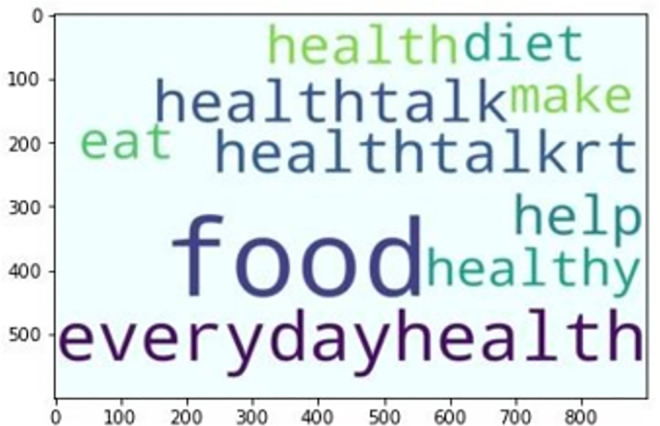
“usnewshealth” top-10.

Instead of measuring independent word combinations [91], the proposed approach aims to achieve a global sentiment polarity of the whole data corpus which merges sources’ heterogeneity, global term relevant frequency and an additional sentiment feature called “subjectivity”. A word-cloud distribution of most frequent words related to healthcare within “everydayhealth”, “gdnhealthcare”, “usnewshealth” is depicted by Figs 14–16 respectively.

#### Polarity vs subjectivity

**Fig 17:**
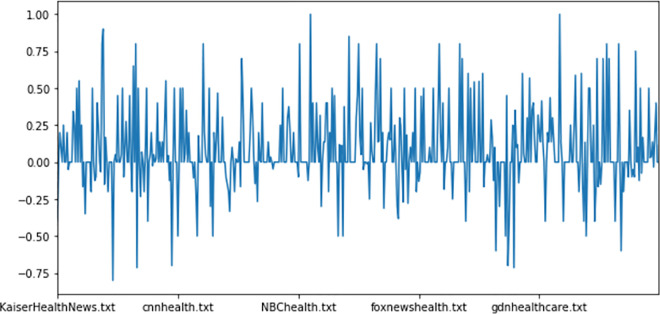
Overall polarity distribution.

In healthcare domain, it is commonly used to detach the sentiment polarity from the sentiment subjectivity [52, 91, 92]. However, as illustrated by Fig 17, it has been found a high correlation between high frequent tokens and their correspondent polarity/subjectivity. The Polar {P} and subjective {S} values are interpreted as follows:

P = {> 0 → Positive sentiment

                0 → Neutral sentiment

                < 0 Negative sentiment}

S = {0 → Objective sentiment

        > 0 → Subjective sentiment}

**Fig 18:**
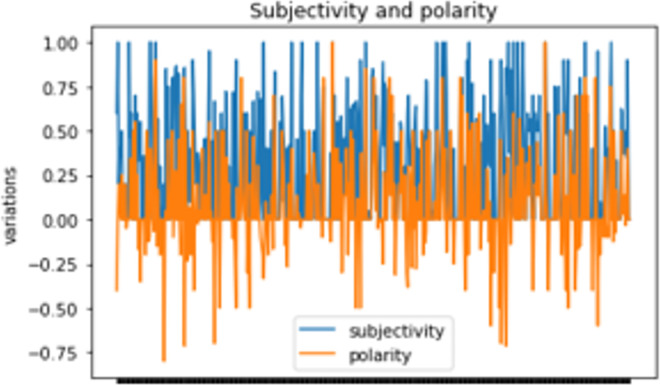
Subjectivity and polarity of tweets.

Figs 17 and 18 show the overall polarity distribution as well as polar/subjective variations respectively of health news tweets based on relevant terms frequency distribution.

**Fig 19:**
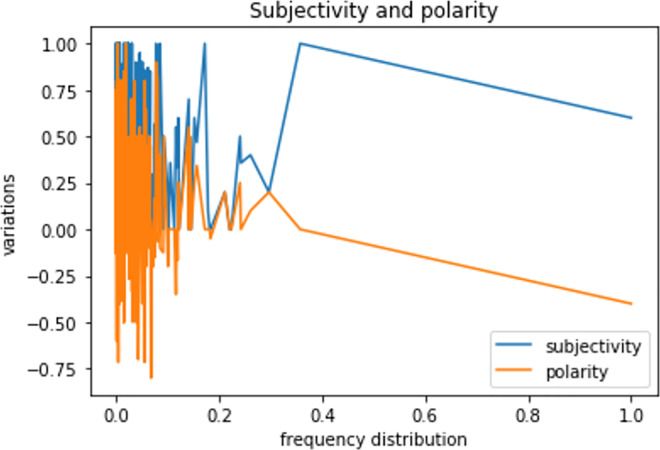
Terms frequency and polarity/subjectivity.

**Fig 20:**
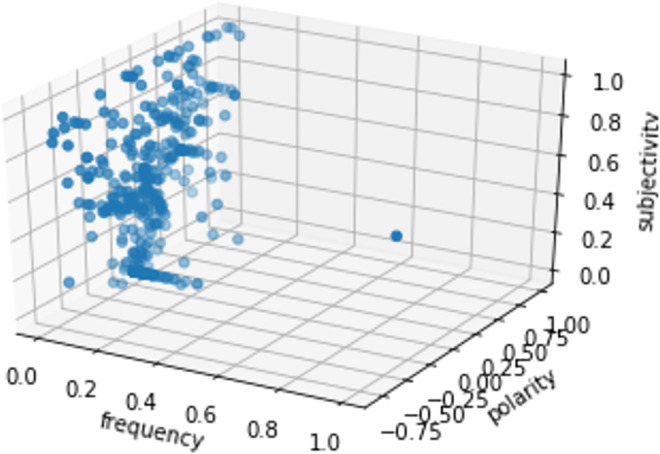
3-d plot frequency, polarity and subjectivity distribution.

Among the 16-health news, only 34.3% of frequent tweets expressed negative healthcare sentiments (P < 0), while 70.4% of them were objective (S < 0.5), this is due to the informal nature of tweets. Furthermore, an interesting observation concerns most frequent terms (Figs 19 and 20) where there was a parallel symmetric decrease of sentiments towards negative and objective feedbacks, which imbalances the overall positivity of tweets as well as their subjectivity.

### Predictive analysis

**Fig 21:**
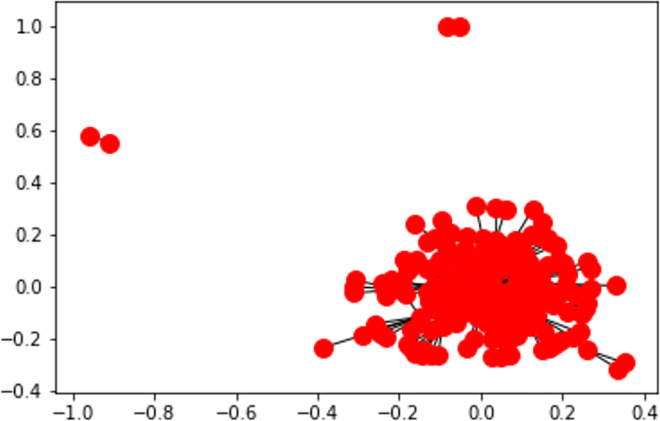
Station-polarity graph generation without edge embedding.

**Fig 22:**
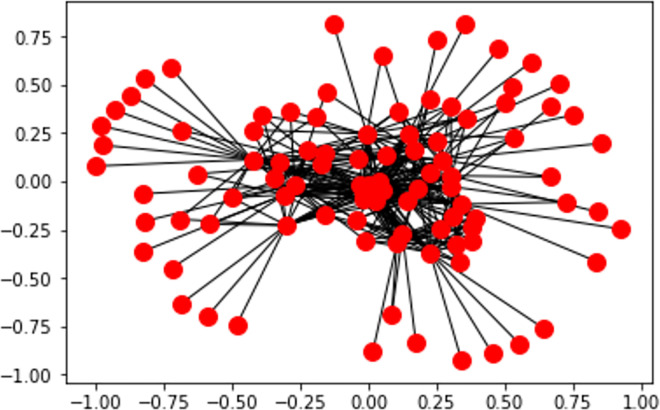
Station-polarity graph generation after edge embedding.

By the proposed model, it is aimed to go beyond the subjectivity or polarity detection, to achieve a transparent predictive analysis of tweets. The goal is to take the above observations over tweets level, but to the data source level. The technique consists of a graph generation which is centred around the 16 health news stations, so given a source of tweets, it would be possible to predict the sentiment polarity/subjectivity instead of going through each tweet, then together these stations are connected within a map (Figs 21 and 22). This application could be seen as community sentiment polar prediction. The following definitions have been proposed to better approach the “Research questions” (RQ3 and RQ5).

**Definition. 1** Given a graph G = (V, E), where a set of tweets’ stations V = {*v*_1_, …,*v*_16_} and a predictable set of edges E = {*e*_1_, …, *e*_*N*_} and N is total number of tweets. A positive sentiment polarity prediction (p) for each station is a link prediction/inference problem where a connection *e*_*i*_ = *v*_*i*_∝*p* exists iff: $\left(\frac{1}{N}\sum_{1}^{N}P({Tweet}_{i})\right)>0$

**Lemma.** Performing edge embeddings on the source data prevents the worst-case iteration (i.e., negative or positive sentiments) and maps the station polarity from DNN prediction to a link prediction problem.

**Fig 23:**
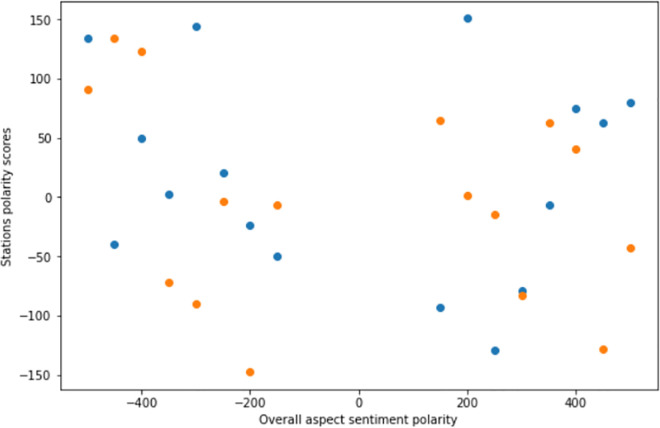
Two dimensions (station-polarity) graph embedding.

**Fig 24:**
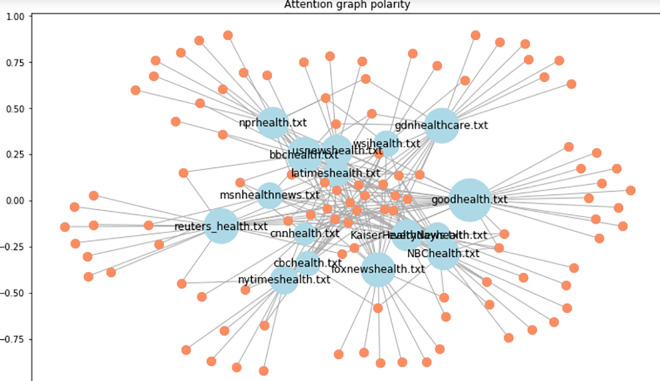
Attention scores for stations’ polarity predictions.

**Example.** The following Figs 23 and 24 represent the sentiment polarity of different stations’ tweets before and after applying edge embeddings respectively.

In addition to the visibility gained by embedding the graph edges, node embeddings (Fig 23) allow a reduced representation of the observed polar sentiments with a clear polar symmetry within the news stations. In our case, the generated graph consists of a set of nodes which are only identified by their labels without any other features. As this is not supported by the recent embedding algorithms (e.g., GraphSage [85]), an abstract version of node2vec algorithm has been implemented which instead of randomly iterates over all connections, it aggregates the neighbourhood nodes of a given station following the predefined constraint (see Definition.1).

**Definition 2.** A scored connection between a station and a sentiment polarity is a neighbourhood aggregation of the scores of its neighbours such as:

$\sum_{1}^{n}p_{i}>0$ (or *any other threshold condition*) needs to be verified during feed-forward and back-propagation stages of the neural network all over the (n) dependencies.

As shown by Fig 24, scoring the positive polarities allows a transparent connectivity as well as inferring new connections.

#### DNN construction

A flexible manner to implement the above steps is to proceed a DNN coding from scratch. With respect to the structure depicted by Fig 12, it has been chosen to use the “Tanh” activation function on the two hidden layers which approximate the sentiment polarity [–1, 1], the output layer has been activated by the “Sigmoid” function which scales the polar vector resulting from hidden layers into positive or negative sentiments, Where:

$$F_{1}(x)=Tanh(x)=\frac{e^{x}-e^{-x}}{e^{x}+e^{-x}}$$ (7)

$$F_{2}(x)=Sigmoïd(x)=\frac{1}{1+e^{-x}}$$ (8)

Table 4 details the parameters of the DNN structure depicted by Fig 12, the batch size of each hidden layer, the activation functions, the optimizer, and the estimated learning rate of each layer.

**Table 4:** Inner structure parameters of the proposed DNN compared to basic techniques.

Index	Input Space Embeddings		DNN configuration			Backward path decoration	Parameters
			Activation function	Optimizer	Batch size		Learning rate
#1	Input Layer	A reduced vector representation	Rectified linear unit (Relu) [93]	Adam	32	• Features already selected and normalized (feedforward embeddings)	-
		• Vector size is given followed users’ rigor around the most frequent term within the text. (results below were performed by selecting 200 neighbourhood nodes)					
						• Weights have been scored and fixed.	
						• (150.2ms of CPU time)	
#2	Hidden layer 1	20 neurones	Tanh	Adam	16	• The performance is partially backpropagated through a set of visited weights.	0.027
						• (573.1.0ms CPU time)	
#3	Hidden layer 2	10 neurones	Tanh	nadam	10	• Skip a given feature while still training if not scored	0.341
						• (721.4ms CPU time)	
#4	Output layer	2 neurones (to predict 2 outputs: positive, negative sentiment.)	Sigmoïd	nadam	8	• Resulted most weighted and scored sentiment is predicted as a major news station polarity.	0.875
						• (310.0sm CPU time)	

As presented by Table 4, the model’s learning increases from thee hidden layers (0.027 to ≈ 0.9) by the output layer, which confirms the hypothesis of the chain rule (Fig 12) (i.e., most of learning happens at the decisional and particularly the output level.). The ReLu activation function has been activating the input layer as it provides better approximation for the embedded features vector, where no classification has made yet except for the frequency analysis (#1 in Table 4), Tanh function has best approximation for sentiment polarity (more detailed on section 6, “DNN construction”). Sigmoid has been activating the output layer to infer positive and negative instances.

**Fig 25:**
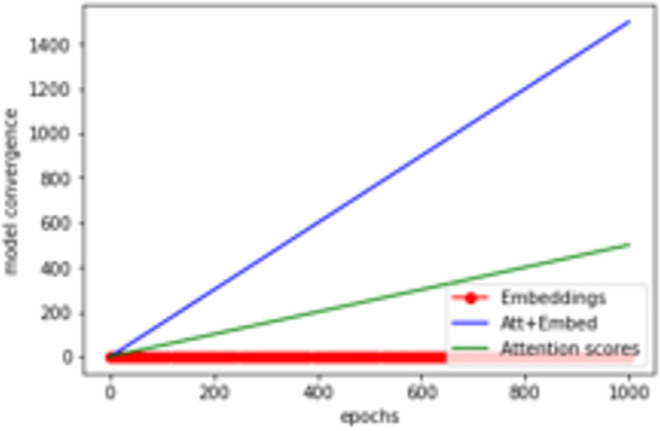
Impact of attention scores and embeddings on the model convergence.

**Fig 26:**
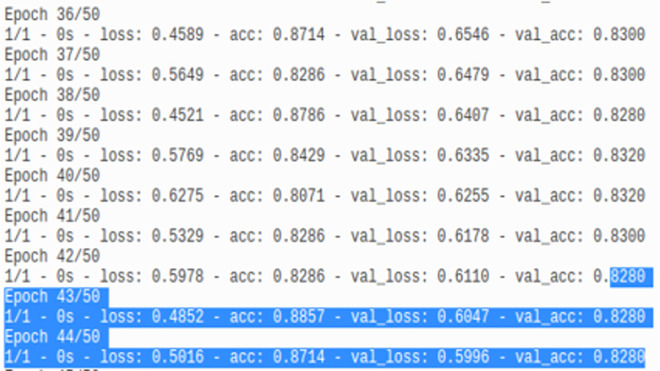
Model loss and accuracy history.

As mentioned by Fig 25 and by displaying the model training history (Fig 26), it has been shown a rapid convergence to a stable accuracy of ≈ 83% which provides an answer on how to stop the model’s vanishing while it keeps propagating even if it reaches an optimal performance.

Table 5 matches the meta-parameters involved within this study with their meaning regarding the studying domain.

**Table 5:** Meaning of the learning metrics’ parameters with regards to the SA study.

Index	Parameters	Signification
#1	True positive sentiment	The model correctly predicts the positive sentiment class (e.g., class of polarity > 0, class of subjective sentiments, subjectivity ≈ 1, etc.).
#2	True negative sentiment	The model correctly predicts the non-existence of an observed fact within a class (e.g., neutral tweets, polarity = 0, etc.)
#3	False positive sentiment	When the model made incorrect predictions about a positive class (i.e., negative polarity detected among most frequent positive terms).
#4	False negative sentiment	In this case, the model incorrectly predicts the negative class (e.g., matching positive frequent term with a class polarity < 0).
#5	Observed population	This term has been used as a reference to all the instances (tweets), covering the four previous defined parameters.
#6	True instance positively predicted	This instance has a similar meaning as the 1^st^ parameter, the difference is a frequent term within a class which is the output instead of the whole class.

- **A*ccuracy*** is the proportion of true results among all the observed population: ***Acc*** = $\frac{truepositive+truenegative}{truepositive+truenegative+falsepositive+falsenegative}$
- ***F-measure*** is the mean between precision and recall: ***F-measure*** = $\frac{2*recall*precision}{recall+precision}$
- ***Precision*** is the proportion of true instances positively predicted among the true positive and false positive identified ones. **Precision** = $\frac{truepositive}{truepositive+falsepositive}$
- ***Recall*** reports the positive polar samples correctly predicted to the total positive samples. It reflects the model’s ability to infer positive samples. **Recall** = $\frac{truepositive}{truepositive+falsenegative}$

The following Table 6 reports the sentiment classification metrics used in this work and the obtained values. We highlight within the same table the impact of the proposed techniques one by one on the model’s performance.

**Table 6:** Proposed model performance (shown with bold) compared to different techniques on health news tweets dataset.

Performance Techniques	Accuracy (%)	Precision (%)	Recall (%)	F1-score (%)
Multi-layer DNN (scratch)	67	51	53	53
Multi-layer DNN + input space embeddings (ISE)	72	68	53	61.4
**Multi-layer DNN + input space embeddings + scoring mechanism**	**≈ 83**	**78**	**89.5**	**83.3**

Due to the features’ opacity, a naive Multi-layer DNN shows a low accuracy (67%) and a poor inference of true instances positively predicted (e.g., 51% precision). However, applying the same technique after excluding the nonrelevant features after graph embeddings (ISE in Table 6) has improved the model’s accuracy as well as the precision, but the recall’s rate remains stable. This is explained by the conditional step (see 2^nd^ part of algorithm above, line 11) where the latter only considered the positive sentiments while the recall implies the positive instances among all population including the negative ones. By coupling the previous step with the scoring technique (a detailed explanation is given in “Improving DNN performance via a deterministic backward walk”), the model has seen a significant improvement among all metrics, that is justified by the determinism gained from selecting relevant features during backpropagation, because this selection covers the activation functions’ derivatives, both positive and negative instances have been covered, thing that explains the recall improvement (from 53% to 89.5%) as well as the other metrics., which answers the second part of “Research questions” (RQ5).

### Complexity analysis

#### Time complexity

The following formula: $\sum_{i=0}^{n}forward\;(activations)+\sum_{i=0}^{n}backward\;(derivatives)$ calculates the overall asymptotic complexity (TC) of a DNN. By considering a given threshold (h), a feed-forward propagation is limited to the input space embeddings times the cost of the activation functions. In our case there are two hidden layers activated with (tanh) and (sigmoid) functions respectively. Suppose:

*TC*(tanh) = *O*(t) and *TC*(sigmoid) = *O*(s), because (tanh) has bigger approximation: *O*(t) > *O*(s)

graph embeddings complexity is *O*(|V|), V is the total graph nodes, therefore:

$TC(\sum_{i=0}^{n}forward\;(activation))=O(\left|V\right|)\cdot O(t)$

For back-propagation, the time complexity is reduced to the scoring method which has (h) as a limit, therefore: *O*(score) = *O*(*V*_*h*_+*E*_*h*_), from that:

*TC = O*(*V*_*h*_+*E*_*h*_)+*O*(|*V*|)⋅*O*(*t*) which may be reduced to *O*(|*V*|)⋅*O*(*t*) in the worst case. The latter reflects the node embeddings strategy adopted by the proposed method.

#### Space complexity

Instead of storing the matrices [94] of feature vectors and parameter weights in memory during the execution of the DNN model, the embedded graph entities are mainly supposed to allocate the memory with the activation function traces. At a time instance epoch(i), (i = 1…90) the proposed model history (e.g., Fig 26) allows to record the following metrics summarized in Table 7.

**Table 7:** CPU occupancy and learning metrics for the proposed model.

	Hidden layer 1			Hidden layer 2			Output layer		
epoch(1)	CPU (%) 92	Acc (%) 41.8	Loss (%) 58.2	CPU (%) 98	ACC (%) 41.8	Loss (%) 58.2	CPU (%) 98	Acc (%) 58.1	Loss (%) 41.9
epoch(16)	71	51.8	48.2				71	68.1	31.9
epoch(90)	51	79.2	20.8	42	80.6	19.4	30	82.9	18

The cache hierarchy of the CPU enables to record several training batches of the proposed DNN (see Table 7). The execution flow shows a reduced footprint (i.e., 3.0 CPU occupancy) resulted from the graph embeddings followed by the backward scoring (see the below section). The reduced instruction vector may represent an alternative to the indeterministic sparsity solution [95] for an efficient DNN training.

**Fig 27:**
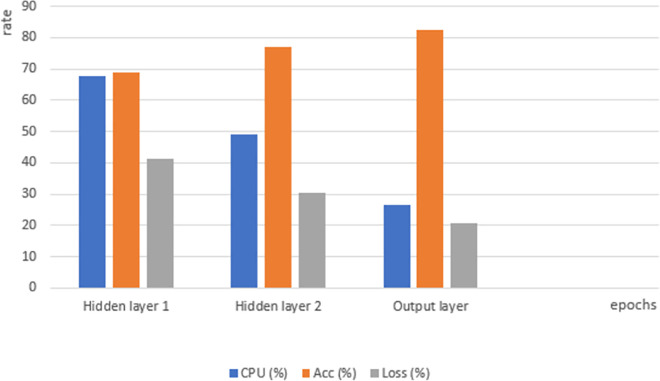
Average CPU time and model’s efficiency through each layer.

As it is shown from Fig 27, the CPU experiences a batch of training and most of its time on the first model’s layers (hidden layers from Fig 27), with an average CPU time of 67.6% in first hidden layer to 49.09% in second one, it ends with less CPU occupation with an average of 26.7% on the decision (output) layer. That justifies our hypothesis about the repetitive work in the input unit of a DNN. However, the model’s accuracy is shown to perform reasonably well since earlier neurones, that’s due to the selection strategy which prevents features’ sparsity and overfitting.

## Evaluation

By this section, the impact of the proposed learning method will be emphasized through different stages: training, learning, complexity and validation.

**Fig 28:**
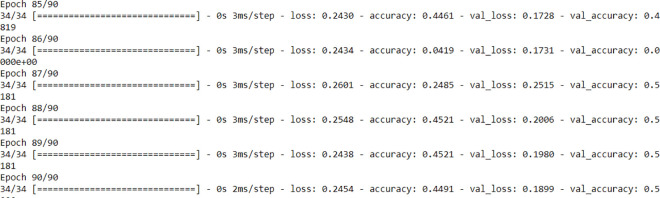
Stability of proposed DNN after 90 epochs and 10 batches.

Due to the heterogeneity of the 16 news’ stations and features’ sparsity imposed to the generated graph components (i.e., nodes are only identified by their labels), the preliminary tests (Fig 28) show a low model performance even if it does not overfit after embedding the input space, the low accuracy remains an issue if not improved, because DNNs are known to perform well with huge data corpus.

**Fig 29:**
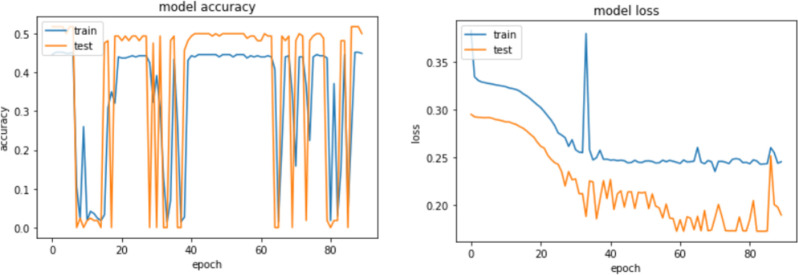
(a) Instability of accuracy. (b) Loss minimization.

Although the loss has been significantly minimized (Fig 29(B)), the instability remarked within the accuracy (Fig 29(A)) variations remains a bottleneck towards the model adaptability.

### Improving DNN performance via a deterministic backward walk

**Fig 30:**
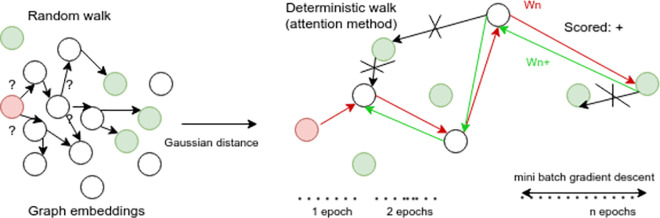
Neural path’s embedding + scoring.

As shown by Figs 25 and 30, scoring the learning path which is recognized while training the DNN model became a mandatory step in our case study, in order to improve the whole accuracy. This will represent a typical example of a good trade-off transparency (graph transparency) and efficiency (DNN performance).

#### Transparency and learning performance

**Fig 31:**
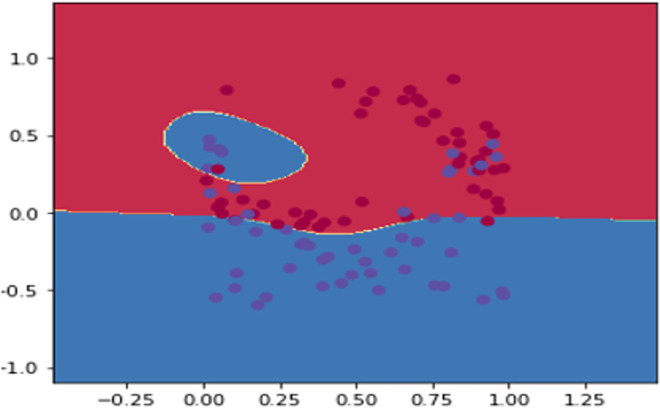
Naïve training and learning.

**Fig 32:**
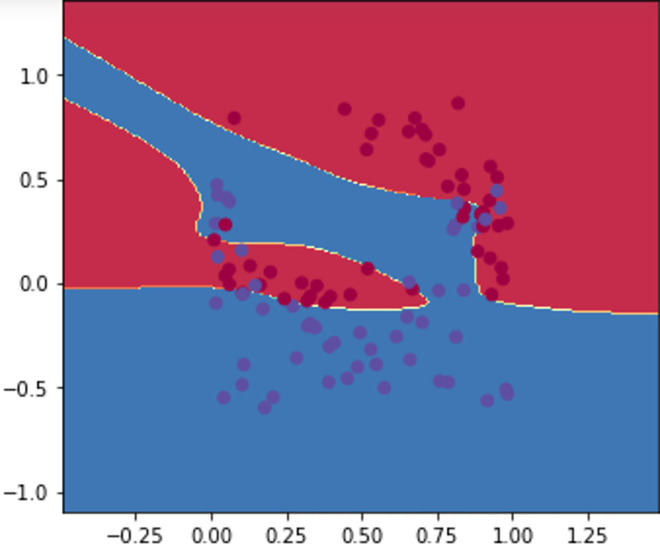
Decision boundary after edge embedding.

**Fig 33:**
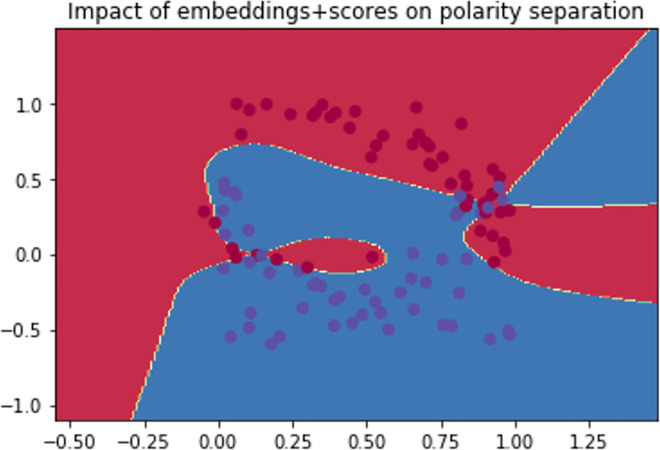
Impact of edge embedding and path scoring on the decision threshold.

The restriction imposed to the input nodes allowed a level of transparency regarding the predictive study, this has been replicated on the feed-forward path, where as described by Figs 31–33, if we consider positive sentiments (polarity) as “blue” instances and the negative ones as “red” ones, the decision boundary showed a better separation of both polarities. However, best adjustment is shown by Fig 33 after scoring the back-propagation path (stamping positive polarity as a constraint).

**Fig 34:**
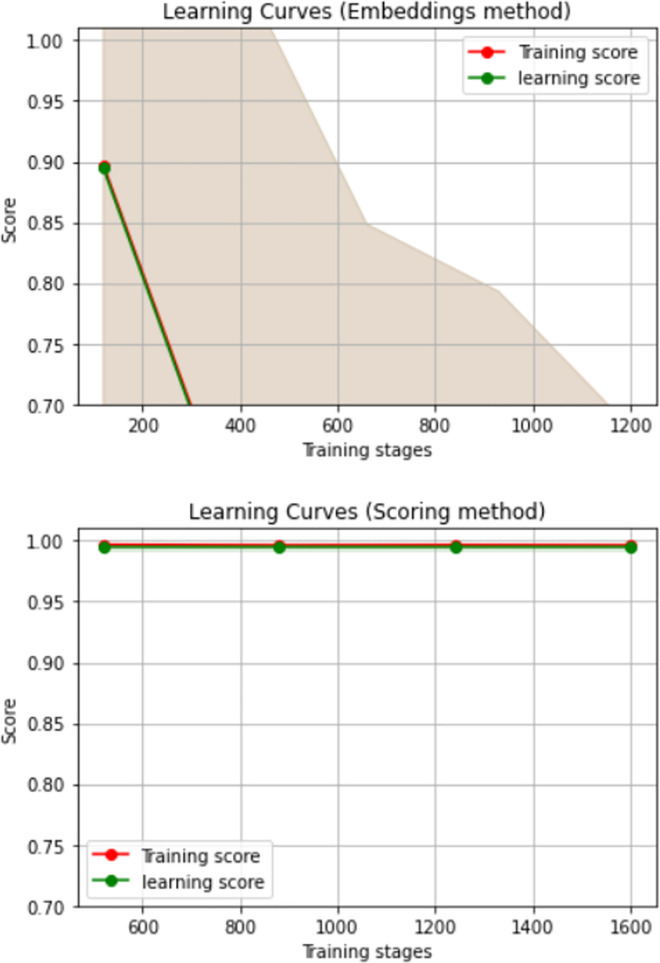
Learning improvements with embedding then scoring techniques.

Consequently, results on adjusting the learning curve with both embeddings and scoring methods sequentially with respect to training scores (batch gradient descent) are illustrated by Fig 34.

**Fig 35:**
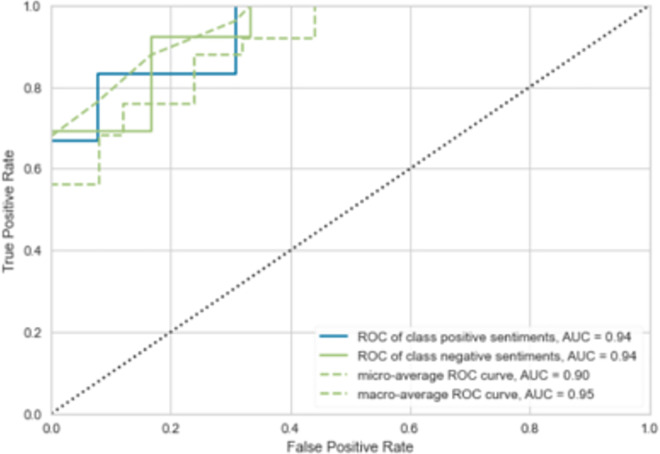
ROC curves for the proposed DNN model.

The Receiver-Operating-Characteristic (ROC) and Area-Under-the-curve (AUC) are two relevant metrics for models’ confidence especially in healthcare domain [96], those two metrics allow to visualize the trade-off between the model’s sensitivity and specificity, where:

- Sensitivity = true-positive rate (rate of correctly identified sentiments)
- Specificity = 1 –false-positive rate (rate of incorrectly identified sentiments)

as illustrated by Fig 35, the proposed learning model showed a higher AUC of 94% with 90% maximization of correctly identified sentiments.

#### Comparing to other methods

As a part of the evaluation, the proposed model is compared to several computational frameworks related to healthcare domain which aimed to analyse tweets and extract sentiment polarity following specific topics. SA was the most targeted topic [97] among the other related domains. However, this process is still not disclosed, and the feature extraction mechanism for sentiment clustering is still not well defined. As depicted by Table 8, common works which have addressed twitter health news dataset used machine learning techniques for sentiments’ classification. However, as argued in the next section, a deep investigation of SA requires different approximations which go beyond linear ML models.

**Table 8:** Comparison of the proposed method (shown with bold) performance with other approaches on twitter health dataset.

Performance Technique	Objective	Acc (%)	Sensi_ tivity (%)	F1-score (%)	Recall (%)	Complexity
• Linguistic enquiry and word-count classifier	Negative tweets detection	74.28	78	80	71	Time and space limit.
• Topic modelling [98]						
• Fuzzy-latent semantic analysis [99]	Topic-based medical documents classification	76.24	70.7	83.7	73.2	O(n),
						n = number of data instances.
• SVM and word embeddings model for tweets indexing	Binary tweets’ classification into self-experienced and non-relevant ones.	81.5	74.1	64.5	70.2	/
• LSTM model for sentiment classification [100]						
• AYLIEN-API ML package for SA [101]	Polarity and subjectivity determination of health tweets	**90** subjectivity/objectivity	**≈**65	78.1	74.2	/
		&				
		≈70 polarity detection				
**DNN implementation:**	**Predictive study to generalize the polar/subjective classification from tweets level to the health news’ sources level.**	**≈ 83**	**79.7**	**89.5**	**75.2**	***O*(|*V*|)⋅*O*(*t*), V is the number of embedded graph nodes, “t” refers to “tanh” function.**
• **Graph Embeddings to reduce input space**						
• **Attention scores for a deterministic backpropagation walk**						

Our proposed method shows great outcomes comparing with other techniques (Table 7), this could be emphasized with the following aspects:

**Semantic enrichment:** our proposed DNN covers both sentiments within separate tweets as well as the whole text corpus for an overall polarity [–1, 1] and subjectivity [0, 1], this includes most frequent terms.

**Complexity:** a complexity analysis has been explicitly conducted, the asymptotic results follow the abstraction strategy (Fig 12) by restricting the whole model complexity to the embedded nodes times the complexity of the decisional function (Tanh). That performance is much better than considering all input space for instance [99].

**Efficiency/determinism:** Although SVM has proven its robustness and performance in many SA tasks (see Table 2), its combination with LSTM represents a bottleneck towards a boosted performance. This could be justified by the pre-training and dependency cost of LSTM at the input data [100]. However, our proposed backpropagation selective strategy increases the model’s determinism (i.e., rapid surge of the learning rate (Fig 34)).

**Transparency:** Our model is characterised by a transparent prediction generation process, this includes the earlier conceptual stages (i.e., Figs 12 and 13) followed by a visual data distribution and the impact of the proposed techniques on best adjusting the decision boundary for sentiment classification (Figs 31, 32 and 33). As opposite to the classical classifiers [102], the proposed DNN structure allows different approximations of the problem (i.e., polarity, subjectivity, frequency, etc), that enables a global observation of the SA over all the news’ stations. The compliance of the backward selection method with backpropagation algorithm (see: “Features’ selection via attention scoring”, “Improving DNN performance via a deterministic backward walk”) does not require any additional training examples or hidden layers as the case in [103], which allowed the model complexity to be restricted to the embedded space.

## Discussions

### Models on explainable AI

**Fig 36:**
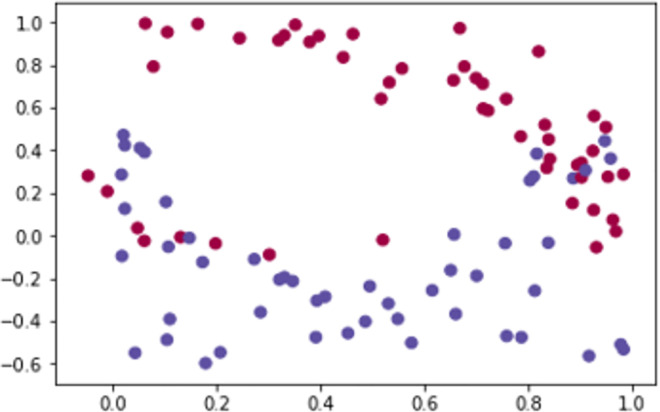
Binary sentiment polarity distribution of tweets.

- Although DARPA’s user interface [74] has been built around users’ expertise and their cognition ability, it disguises the traceable aspect of the prediction making, which may include the active neurons and the prediction path.
- Instead of explaining learning models after their realization, current trends in machine learning [104] suggest that it is more prominent to include explicability from the first conceptual steps of the model. However, as illustrated by Fig 36, the non-linear distribution which results from distinctive feature scales (e.g., Frequency [0…n], subjectivity [0…1], etc.) requires an alternative method than traditional nonlinear ML approximation, where the latter is applied to the whole observations. A DNN could approximate each feature observation following specific layers, that what explains a higher sensitivity and recall performance (Table 8).
- LSTM can only relate a given aspect to the previous one. But within the SA context, further dependencies may occur and need to be captured. For instance, in [100] (see Table 8) an index had to be done in order to boost the model performance.
- A good understanding of the input dataset could be achieved by an efficient pre-processing. However, with DNNs, this does not guarantee a good performance, as the latter (see 21) is usually conditioned by a random weight assignment to activate certain functions. By the proposed model, we aim to make this process more deterministic.
- Data is usually pre-processed before trained and validated by a DL model, that helps removing impurities like stop words, insignificance, etc., but eventually promote the loss of data information centrality. Whereas, by investigating a graph theory (i.e., embeddings) accompanied with a DNN data closeness centrality is preserved (Fig 23).

### Limits

- Although the proposed model showed great convergence which prevents vanishing problem and saves training time, its performance was relatively weak when deployed on x86 architecture with 5 GB available RAM (Fig 28).
- The embedding method prevents the DNN to broad the learning scale because the layers are activated by proceeding the embedded vector although the model backpropagates through all the instances (see Algorithm above) even though the loss measure is considerably less (Fig 29(B)), it mainly optimizes the scored weights (e.g., positive weights).
- Disclosing features semantics in [99] has proven its resiliency in handling unstructured data. In our model, the embedded feature vector as well as the scored samples could be enriched by an accompanied context vector for understandability purposes.

## Conclusion and future work

In this research work, we aim to propose a transparent DNN model for a sentiment classifier. It has been decided to proceed the development without using built-in DL libraries except for evaluation metrics invocation, and that was in order to exactly design each unit: input, decision and output with the defined method (see “Methods”). The latter consists of a new performance improvement strategy which combines a sparse graph embedding (i.e., node, edges with no features) and scoring paths for the input and decisional units respectively. The model is trained and tested on Twitter health news dataset, where a sentiment predictive analysis has been applied to each news sources based on the most frequented tweets. We broad the feature space by normalizing both token aspects and tweets for each of the 16 news so that a global sentiment polarity is inferred. Results show state-of-the-art performance while comparing to other models (see “Predictive analysis” and “Comparing to other methods”). Moreover, the transparency and the efficiency of the model in stabilizing the learning curve with better binary classification of tweets (see above).

This work can benefit from several improvements in the future. For instance:

- Exploring the transferable learning aspect of graph embeddings to include other updated topics on twitter (e.g., Covid-19) where more transparency is required. This may be achieved by moving from the transductive to the inductive learning. Furthermore, that may provide an answer to the dynamic aspect of graphs as the input data may evolve over the time.
- Proving the model resiliency against new unstructured and semi-structured data (SemEval-2014 task7 [105]).
- In terms of performance, it has been proven that the embedding technique had a big impact on the model accuracy (see “Evaluation”). Thus, by considering a context features’ vector while training the model, this could broad the learning stage and improve the model performance.
